# RetroCaptioner: beyond attention in end-to-end retrosynthesis transformer via contrastively captioned learnable graph representation

**DOI:** 10.1093/bioinformatics/btae561

**Published:** 2024-09-28

**Authors:** Xiaoyi Liu, Chengwei Ai, Hongpeng Yang, Ruihan Dong, Jijun Tang, Shuangjia Zheng, Fei Guo

**Affiliations:** School of Chinese Materia Medica, Beijing University of Chinese Medicine, Beijing, 102488, China; Ministry of Education, Engineering Research Center for Pharmaceutics of Chinese Materia Medica and New Drug Development, Beijing, 100102, China; Computer Science and Engineering, Central South University, Changsha, 410083, China; Computer Science and Engineering, University of South Carolina, Columbia, South Carolina, 29208, United States; Academy for Advanced Interdisciplinary Studies, Peking University, Beijing, 100871, China; Faculty of Computer Science and Control Engineering, Shenzhen University of Advanced Technology, Shenzhen, 518055, China; Shenzhen Institute of Advanced Technology, Chinese Academy of Sciences, Nanshan, 518055, China; Global Institute of Future Technology, Shanghai Jiao Tong University, Shanghai, 200240, China; Computer Science and Engineering, Central South University, Changsha, 410083, China

## Abstract

**Motivation:**

Retrosynthesis identifies available precursor molecules for various and novel compounds. With the advancements and practicality of language models, Transformer-based models have increasingly been used to automate this process. However, many existing methods struggle to efficiently capture reaction transformation information, limiting the accuracy and applicability of their predictions.

**Results:**

We introduce RetroCaptioner, an advanced end-to-end, Transformer-based framework featuring a Contrastive Reaction Center Captioner. This captioner guides the training of dual-view attention models using a contrastive learning approach. It leverages learned molecular graph representations to capture chemically plausible constraints within a single-step learning process. We integrate the single-encoder, dual-encoder, and encoder–decoder paradigms to effectively fuse information from the sequence and graph representations of molecules. This involves modifying the Transformer encoder into a uni-view sequence encoder and a dual-view module. Furthermore, we enhance the captioning of atomic correspondence between SMILES and graphs. Our proposed method, RetroCaptioner, achieved outstanding performance with 67.2% in top-1 and 93.4% in top-10 exact matched accuracy on the USPTO-50k dataset, alongside an exceptional SMILES validity score of 99.4%. In addition, RetroCaptioner has demonstrated its reliability in generating synthetic routes for the drug protokylol.

**Availability and implementation:**

The code and data are available at https://github.com/guofei-tju/RetroCaptioner.

## 1 Introduction

Retrosynthesis is a fundamental strategy in organic chemistry, crucial in drug discovery and chemical biology (Zheng *et al.* 2022), aiming at constructing compounds through various organic reactions (Zhong *et al.* 2023). In drug discovery, retrosynthesis assists chemists in deconstructing novel drugs into simpler, commercially available, or easily synthesizable precursors, guiding the design of synthesis routes (Mallick and Bhadra 2023). In the early stage, retrosynthesis models primarily aimed at predicting optimal templates, utilizing these templates to convert products into reactants (Coley *et al.* 2017).

In recent years, with the rapid development of deep learning technologies, many approaches (Chen *et al.* 2020, 2023b) have been proposed to enhance retrosynthetic capabilities and make the design process of synthetic experiments more efficient without the template. Existing machine learning-based models can be roughly divided into two categories: sequence-based and graph-based methods regarding molecular representations (Meng *et al.* 2023). Although current state-of-the-art models represent molecules as graphs (Wang *et al.* 2023), which are used to predict changes in the target molecule. This is usually done through a multi-stage paradigm that involves synthon completion with the prediction of matched leaving groups. However, they are limited to datasets focusing on the pre-collected Leaving Groups (LGs) decomposed from the specific dataset (Wang *et al.* 2023). Recently, there has been a significant rise in the development of language models (Liu *et al.* 2023). Consequently, retrosynthesis has been reconceptualized as an end-to-end translation task, where molecules are represented as text, such as Simplified Molecular Input Line Entry System (SMILES) strings, to translate products into reactants (Weininger 1988, Wang *et al.* 2021, Ucak *et al.* 2022).

With the development and practicality of language models, the Transformer architecture (Vaswani *et al.* 2017) has established its superiority in machine translation tasks with self-attention mechanisms. For instance, Zheng *et al.* (2019) proposed the SCROP model, which is built on a Transformer-based predictor and a multi-head attention Transformer grammar corrector. This corrector is designed to fix syntax errors, thereby enhancing the chemical validity of predicted outputs. To better integrate chemists’ retrosynthetic strategy, Wang *et al.* (2021) introduced RetroPrime, a model also using two Transformers to identify reaction centers and convert molecules into reactants through synthons. However, relying solely on linear SMILES representations overlooks the structural information present in molecular graphs. Besides, such a method (Wang *et al.* 2021) requires training two separate modules to complete the transformation, and the efficiency needs improvement.

To fully exploit the structural information of molecules, several Transformer variants have been introduced (Tetko *et al.* 2020, Kim *et al.* 2021, Seo *et al.* 2021, Wan *et al.* 2022). Seo *et al.* (2021) proposed the GTA model, which masks the self-attention layer using the adjacency matrix of the product molecule in the encoder and applies the cross-attention layer in the decoder to capture atomic correspondence between reactants and products. Tu and Coley (2022) presented Graph2SMILES, which uses a sequential graph encoder. Its comparable performance underlines the significance of enhanced graph representation. While existing methods using molecular graphs (Lin *et al.* 2023, Xie *et al.* 2022) have achieved remarkable performance, their efficiency is hindered by the two-step process that requires training two separate modules. These methods are also limited due to the restricted scope of the constructed leaving group datasets (Zhong *et al.* 2023). Since the module for predicting LGs is designed to make predictions on a pre-collected LGs database, these methods have limited flexibility in generating rare LGs (Wang *et al.* 2023). Given the limitations of graph and SMILES representations, an optimal strategy is needed to effectively capture reaction mechanisms and molecular information (Xiong *et al.* 2019).

Since a reaction center represents the group of atoms and bonds contributing to the chemical transformation (Shi *et al.* 2020, Wan *et al.* 2022), various methods have been proposed to capture this chemically plausible information, including the previously mentioned two-step models (Xie *et al.* 2022). Such methods ignore a strong link between center identification and synthon completion in chemical reactions. Wan *et al.* (2022) introduced Retroformer, which uses a local-global self-attention in the encoder to efficiently combine structural-sequence data, and balance the reaction center with the global context. However, reaction centers can vary: different products have unique reaction centers, and even the same product can present different centers in various reactions (Chen *et al.* 2023b). Thus, this variability underscores the need for a flexible weight allocation in attention models. In contrast, MEGAN (Sacha *et al.* 2021) applies a sequence of edits to the product graph, but the complexity of long edit sequences limits its performance.

**Figure 1.**
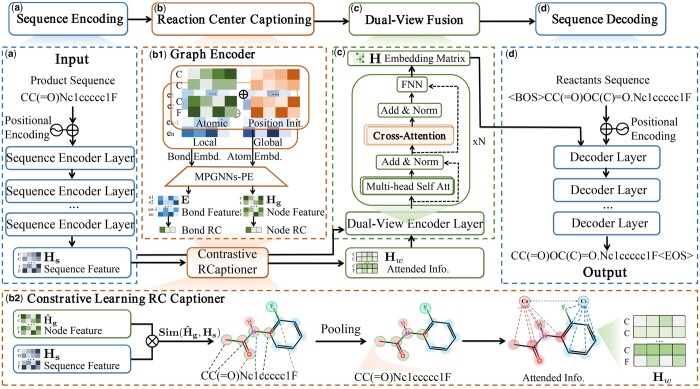
Overview of RetroCaptioner encoder–decoder architecture. This framework comprises four modules. (a) Sequence encoder module. Molecular SMILES embedding with traditional Transformer encoder. (b) Reaction Center captioner (RCaptioner). The RCaptioner used contrastive learning for caption differentiation between RC and non-RC subgraphs, refining atom features based on attention. (c) Dual-view fusion. It utilizes a cross-attention layer to incorporate structural features. The concatenation of representations of two encoder modules is output as the graph representation and input into the molecule decoder as the key and value. (d) Sequence decoder. The decoder auto-aggressively predicts the next token of the molecular sequence.

Herein, we propose RetroCaptioner, a novel end-to-end retrosynthesis prediction architecture. Specifically, RetroCaptioner unifies single-encoder, dual-encoder, and encoder–decoder paradigms with the proposed Contrastive Reaction-Center (RC) Captioner (RCaptioner). RetroCaptioner combines SMILES and graph representations to efficiently capture RC information in molecules. It addresses both long-range characteristics and specific structures like rings, which were inspired by Zhu *et al.* (2023). The RCaptioner allocation of weights for the variability of reactive centers provides a chemically plausible constraint that guides the training of the attention model through contrastive learning. The overall framework of RetroCaptioner is shown in Fig. 1. The contributions are generalized as follows:

1. We present RetroCaptioner, an advanced end-to-end framework featuring a Contrastive Reaction Center Captioner. This captioner guides the training of dual-view attention models through a contrastive learning approach, using learned molecular graph representations to capture chemically plausible constraints within a single-step learning process.
2. We unify the single-encoder, dual-encoder, and encoder–decoder paradigms to effectively combine information from the sequence and graph representations of molecules. This is accomplished by adapting the Transformer encoder into a uni-view sequence encoder and a dual-view module. In addition, we improve the captioning of atomic correspondence between SMILES and graphs.
3. Our proposed method, RetroCaptioner, achieves outstanding performance with 67.2% in top-1 and 93.4% in top-10 exact matched accuracy on the USPTO-50k dataset. It also demonstrates an exceptional SMILES validity score of 99.4%. Furthermore, RetroCaptioner has proven to be reliable in generating synthetic routes for the drug protokylol.

## 2 Materials and methods

### 2.1 Dataset

We evaluate our method using the retrosynthesis benchmark dataset USPTO-50k, grouped into 10 reaction classes from Wan *et al.* (2022). Moreover, following previous research (Wan *et al.* 2022, Zhong *et al.* 2022), we also use SMILES alignment with atom-mapping training (Zhong *et al.* 2022) (see Supplementary Section S2). When training the model, we can obtain multiple input-output pairs as the training data by enumerating different atoms as the root of SMILES (Tetko *et al.* 2020, Seo *et al.* 2021). You can find the details of how to make model predictions with data augmentation in Supplementary Section S3.

### 2.2 Problem description and model construction

A retrosynthesis reaction can be represented as a transformation of a product molecule to a set of reactant molecules (Meng *et al.* 2023). For example, the SMILES string of a reaction can be described as “[RX_2]CC(=O)OC(C)=O.Nc1ccccc1F ≫ CC(=O)Nc1ccccc1F,” where “CC(=O)Nc1ccccc1F” is the product (target) sequence, “CC(=O)OC(C)=O.Nc1ccccc1F” are the reactants sequence, multiple SMILES strings can be concatenated by a full stop “.” into one single SMILES sequence and “[RX_2]” is the reaction class 2. Following Zheng *et al.* (2019), each reaction was split into reactants and target sequences for model training. Thus, for the retrosynthesis prediction task, given a product molecule M, the target is to predict a set of *N* reactant molecules ${\left\{M_{i}^{R}\right\}}_{i=1}^{N}$ that can lead to M.

### 2.3 RetroCaptioner encoder–decoder architecture

The RetroCaptioner framework, illustrated in Fig. 1, leverages an optimized end-to-end, Transformer-based architecture for one-step retrosynthesis It comprises four main modules: (i) uni-view sequence encoder, (ii) contrastive reaction center captioner (RCaptioner), (iii) dual-view sequence-graph encoder, and (iv) Transformer decoder. Unlike traditional encoder–decoder Transformers (Vaswani *et al.* 2017), RetroCaptioner uses a Transformer encoder specifically for uni-view sequence representations and cascades the remaining layers into a dual-view encoder. These layers cross-attend to the molecular graph information, ensuring comprehensive graph-sequence representations. The novel RCaptioner ensures detailed inter-atomic representations with molecular SMILES and graphs. Importantly, RCaptioner introduces a chemically plausible constraint that directs attention model training through a contrastive learning approach, emphasizing the differences in the weights assigned to RC nodes versus non-RC nodes.

#### 2.3.1 Uni-view sequence encoder

The Transformer (Vaswani *et al.* 2017), has proven effective for retrosynthesis prediction tasks (Ishiguro *et al.* 2020, Ucak *et al.* 2022). Consequently, we used the first half of the Transformer encoder as a uni-view sequence encoder (Fig. 1a). With the molecular SMILES sequence $S=\{s_{1},s_{2},\ldots ,s_{L}\}$, where *L* denotes the total length, the sequence encoder output $H_{s}\in R^{L\times d}$ from the input SMILES embedding X by applying the fundamental self-attention mechanism of the Transformer. RetroCaptioner also includes a SMILES alignment task, akin to machine translation. Since many molecules stay unchanged in reactions, graph node alignment (atom mapping) easily translates into SMILES token alignment [see Wan *et al.* (2022) for details].

#### 2.3.2 Contrastive reaction center captioner

In our study, the proposed RCaptioner consisted of two major modules (Fig. 1b): a graph encoder and a contrastive learning captioning workflow. We utilize the Message-Passing GNNs with the Positional Encoding (MPGNN-PE) strategy proposed by Dwivedi *et al.* (2023) to enrich graph embeddings. Concurrently, our RCaptioner contrastively captions the reactive center and learns an optimal molecular graph representation. It integrates the SMILES sequence to capture information from long-distance nodes in the molecular structure.

For graph encoder (Fig. 1b.1), MPGNN-PE (Dwivedi *et al.* 2023) can learn positional representation with Positional Encoding (PE) of nodes and inject it into the input layer. In RetroCaptioner, a molecular graph G=(V,E), are constructed from SMILES strings using the RDKit package (Landrum 2016), where V and E represent the set of *m* atom nodes and *n* edges, respectively (see Supplementary Table S1). For each node *i*, initial atom features $h_{i}^{\mathrm{init}}\in R^{d_{v}}$ (e.g. atoms, charges) where $d_{v}$ is the number of atom features, and the initial feature of PE $p_{i}^{\mathrm{init}}\in R^{k}$ is computed using *k*-steps of the random walk diffusion process [for details, see Dwivedi *et al.* (2023)]. At the layer l=0, the node feature and PE feature are formulated by applying Feed-Forward Network (FFN), $h_{i}^{0}=\text{FFN}([h_{i}^{\mathrm{init}};p_{i}^{\mathrm{init}}])$ and $p_{i}^{0}=\text{FFN}(p_{i}^{\mathrm{init}})$.

In bond embedding, both local and global features are integral. The local feature is characterized by the initial bond feature, $e_{\mathrm{ij}}^{\mathrm{init}}\in R^{d_{e}}$ (e.g. bond type, conjugated). The global feature is denoted by the distance $d_{ij}$ between nodes *i* and *j*, calculated using the 3D conformation optimized by MMFF (Halgren 1996) with Radial Basis Function (RBF), thus, the bond feature is $e_{\mathrm{ij}}^{0}=\text{FFN}(e_{\mathrm{ij}}^{\mathrm{init}})+\text{RBF}(d_{\mathrm{ij}})$ [refer Halgren 1996)], at the layer l=0. The layer update equations are given in Equation (1). Leveraging node and edge features, we adopt strategies akin to Wan *et al.* (2022), utilizing two FFN to detect reaction centers, boosting the performance. Crucially, with end-to-end training of RetroCaptioner, generative feedback can backpropagate to the reaction center learning (see details in Supplementary Section S4).


(1a) $$h_{i}^{l+1}=f_{h}\left(\left[\begin{array}{c} h_{i}^{l}\\ p_{i}^{l} \end{array}\right],{\left\{\left[\begin{array}{c} h_{j}^{l}\\ p_{j}^{l} \end{array}\right]\right\}}_{j\in N_{i}},e_{\mathrm{ij}}^{l}\right),$$

(1b) $$\begin{array}{c} e_{\mathrm{ij}}^{\mathrm{l+1}}=f_{e}\left(h_{i}^{l},h_{j}^{l},e_{\mathrm{ij}}^{l}\right), \end{array}$$

(1c) $$\begin{array}{c} p_{i}^{\mathrm{l+1}}=f_{p}\left(p_{i}^{l},{\left\{p_{j}^{l}\right\}}_{j\in N_{i}},e_{\mathrm{ij}}^{l}\right), \end{array}$$

where $f_{h}$, $f_{e}$, and $f_{p}$ are functions with learnable parameters, and $N_{i}$ is the neighborhood of the node *i*. The output of MPGNN-PE included the final embeddings of nodes and edges denoted as $H_{g}\in R^{m\times d}$ (*m* represents node number) and $E\in R^{n\times d}$ (*n* represents edge number). *d* represents the embedding dimension.

Accurate prediction of the reaction center is meaningful for retrosynthesis. In our framework, we decompose the prediction of response centers into two sub-tasks of nodes and edges prediction. The node and edge features ($H_{g}$ and E), obtained from MPGNN-PE, are used in two FFN identified as the Atom RC Identifier and Bond RC Identifier.


(2a) $$P_{rc}\left(h_{i}\right)=\sigma \left(FFN_{\text{atom}\;}\left(h_{i}\right)\right),$$

(2b) $$\begin{array}{c} P_{rc}\left(e_{\mathrm{ij}}\right)=\sigma \left({\mathrm{FFN}}_{\text{bond}\;}\left(e_{\mathrm{ij}}\right)\right), \end{array}$$

where $h_{i}$ and $e_{\mathrm{ij}}$ are the embedding for node *i* and edge between node *i* and *j*, the scores $P_{rc}\left(h_{i}\right)$ and $P_{rc}\left(e_{\mathrm{ij}}\right)$ represent the likelihood of the node and edge in the graph being predicted as a reaction center.

Furthermore, RCaptioner is capable of capturing the RC information contained among even distant atoms and specific structures by integrating SMILES sequence and graphs (Fig. 1b.2). This is inspired by image-text matching tasks in computer vision studies (Yu *et al.* 2022, Chen *et al.* 2023a) and the dual-view network concept (Zhu *et al.* 2023). Such as Chen *et al.* (2023a) proposed the cross-modal attention mechanism which includes Contrastive Content Re-sourcing and Contrastive Content Swapping constrains for the image-text matching task. Here, we first project the $H_{g}$ into ${\hat{H}}_{g}$ by an FFN projection and divide the nodes into two groups by the known node labels: RC nodes ${\hat{H}}_{rc}\in R^{u\times d}$ or non-RC ${\hat{H}}_{\mathrm{nrc}}\in R^{v\times d}$, where *u* and *v* represent the node number of two groups (u+v=m). $\hat{h}rc^{i}$ and ${\hat{h}}^{j}nrc$ represent the node embeddings for these two groups. As detailed in Equation (3b), the interaction between each node in the graph and each token in the sequence is evaluated by a similarity function, denoted as Sim(·). Subsequently, an average pooling layer Pooling(·), is used to determine the relative significance of each graph node in the context of the full sequence. This process influences the graph embeddings $H_{g}$, as detailed in Equation (3c).


(3a) $${\hat{H}}_{g}=\mathrm{FFN}(H_{g}),$$

(3b) $$\begin{array}{c} w=\text{softmax}(\text{Pooling}(\text{Sim}({\hat{H}}_{g},H_{s}))), \end{array}$$

(3c) $$\begin{array}{c} H_{w}=w⊗H_{g}, \end{array}$$

where $w\in R^{m\times 1}$ is the weight of nodes, ⊗ is element-wise production, $H_{w}\in R^{m\times d}$ represent the weighted graph embedding by the attended information, measured by the full sequence and the latent nodes embedding.

**Figure 2.**
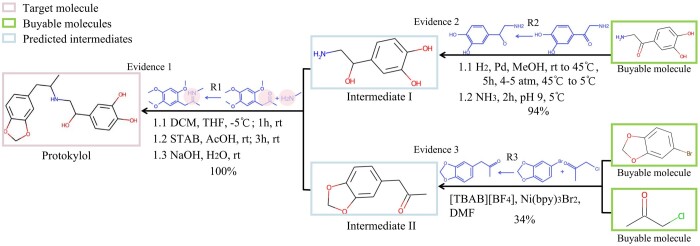
Retrosynthesis planning for protokylol. A two-step synthetic route to protokylol is presented. In every step, the text highlighted in blue indicates the documented evidence that supports the reaction.

Recognizing the variances of reaction centers among various reactions and molecules (Chen *et al.* 2023b). RCaptioner captioning the reactive center and differentiating the attention weights between RC nodes and non-RC nodes through contrastive learning. In detail, two learnable vectors ($c_{\mathrm{rc}}$ and $c_{\mathrm{nrc}}$) are corresponding centers in the graph embedding space. Through contrastive learning, RCaptioner directs attention model training, enhancing traditional attention mechanisms. It combines the two-stage processes methods (Wang *et al.* 2021) into one-step learning by implementing chemically plausible constraints, utilizing the contrastive loss in Equation (4):


(4) $$\begin{array}{c} L_{\mathrm{RCaptioner}}=||c_{\mathrm{nrc}}-c_{\mathrm{rc}}+\gamma |{|}_{2}^{2}\\ +\frac{1}{u}\sum_{i}^{u}||{\hat{h}}_{rc}^{i}-c_{\mathrm{rc}}|{|}_{2}^{2}\\ +\frac{1}{v}\sum_{j}^{v}||{\hat{h}}_{\mathrm{nrc}}^{j}-c_{\mathrm{nrc}}|{|}_{2}^{2}, \end{array}$$

the loss function has three parts: one reflects the distance between center embedding, regulated by γ, and the others penalize deviation from respective embedding centers. This minimization guides the latent embeddings of node groups closer to their centers and widens the center-to-center distance.

#### 2.3.3 Dual-view fusion

Through the RCaptioner with contrastive learning, the model distinguishes between groups, enhancing the differentiation in attention between RC and non-RC nodes. Thus, with the improved molecular representation named Attended info., the dual-view encoder proposed to integrate both SMILES and graph embedding. To achieve this, we used a dual-view fusion module that integrates graph information into the sequence via cross-attention mechanism (Fig. 1c). Each dual-view encoder layer has two attention blocks. The first is self-attention Q, K, and V are based on the outputs of the previous layer. The second is the cross-attention block utilized for the dual-view fusion strategy, where the weighted graph embedding $H_{w}$ will be projected to key and value, and the output of the last self-attention Z are projected to query. The cross-attention is calculated:


(5) $$\text{Attn}(Z,H_{w})=\text{softmax}(\frac{Q^{\prime }K^{\prime T}}{\sqrt{d}})V^{\prime },$$

where $Q^{\prime }=ZW_{Q^{\prime }}$, $K^{\prime }=H_{w}W_{K^{\prime }}$ and $V^{\prime }=H_{w}W_{V^{\prime }}$ and $W_{Q^{\prime }}$, $W_{K^{\prime }}$ and $W_{V^{\prime }}$ are learnable parameters. The output $H\in R^{L\times d}$ of multiple dual-view encoder layers will represent the product, which can efficiently integrate information from graph and sequence.

#### 2.3.4 Decoder

We use a Transformer-based autoregressive decoder to decode from the atom representations H after the dual-view encoder (Fig. 1d). The model aims to match the predicted sequence $Y=[y_{1},\ldots ,y_{k}]$ with the actual reactants sequence $T=[t_{1},\ldots ,t_{k}]$ by minimizing their differences (Tu and Coley 2022).

### 2.4 Loss function and training

The training schema can be viewed as a multitask learning with overall loss as Equation (6):


(6) $$L=L_{LM}+L_{AG}+L_{CL}+L_{\mathrm{RCaptioner}},$$

where $L_{LM}$ is the language modeling objective by using Negative Log-Likelihood (NLL) loss, $L_{CL}$ is a cross-entropy loss for reactive center prediction, $L_{AG}$ is the SMILES alignment loss, and the $L_{\mathrm{RCaptioner}}$ is the contrastive learning loss.

## 3 Results

### 3.1 Evaluation metrics

In evaluating the performance of the single-step retrosynthesis prediction task. We utilized the commonly used evaluation performance metrics top-k (*k* =1,3,5,10) exact match accuracy to evaluate the retrosynthesis performance, which adopted by sequence- and graph-based retrosynthesis methods (Zheng *et al.* 2019, Seo *et al.* 2021, Wan *et al.* 2022, Chen *et al.* 2020, 2023b). The exact match accuracy is computed by comparing predicted reactants SMILES to the dataset’s ground truth on the benchmark dataset [Equation (7)]. We also evaluate the top-k validity of the generated routes. For molecule validity, we treat a candidate as valid if RDKit can successfully identify the molecule SMILES. The top-k validity is calculated as Equation (8):


(7) $${\text{ACC}}_{\mathrm{Top}(k)}=\frac{\text{Sgn}(\sum_{j=1}^{k}I({\hat{y}}_{j},y_{\mathrm{true}}))}{N}\times 100\%,$$

(8) $$\text{Valid}(k)=\frac{1}{N\times k}\sum_{i=1}^{N}\sum_{j=1}^{k}1,$$

where the mathematical Sgn function returns an integer value indicating the sign of the argument. The syntax is Sgn(number), where the number parameter can be any valid numerical expression. The return value is such that if the number is greater than 0, then Sgn returns 1; if it is equal to 0, it returns 0. The function I denotes whether two input strings are equal. It returns 1 if they are equal, and 0 if they are not equal.

### 3.2 Implementation details

RetroCaptioner has 4 uni-view sequence encoder layers, 4 dual-view encoder layers, and 8 decoder layers, the node and edge dimensions in GNN are 128, and the number of steps *k* is set to 20 in the random walk diffusion process. The model is trained with the Adam optimizer (Kingma and Ba 2014) using a fixed learning rate of 1e−3, and a dropout rate of 0.3 (see Supplementary Table S2 for variable dimensions). The embedding dimension is 256, the self-attention and cross-attention heads are set to 8. Supplementary Table S1 shows the bond features used in RetroCaptioner. All experiments were conducted on NVIDIA GeForce RTX 4090.

### 3.3 Comparison with state-of-art methods

RetroCaptioner was compared against the Transformer-based method, including vanilla Transformer (Vaswani *et al.* 2017), SCROP (Zheng *et al.* 2019), Tied Transformer (Kim *et al.* 2021), Aug. Transformer (Tetko *et al.* 2020) and graph enhanced method, such as GTA (Seo *et al.* 2021), GET (Mao *et al.* 2021), MEGAN (Sacha *et al.* 2021), Graph2SMILES (Tu and Coley 2022), RetroPrime (Wang *et al.* 2021), Retroformer (Wan *et al.* 2022), and the graphormer-based (Ying *et al.* 2021) model RetroExplainer (Wang *et al.* 2023) as strong baseline models for comparison. We do not include the pretraining approach in the performance comparison. Therefore, the results of RetroCaptioner are also comparable to those graph-based models, offering improved efficiency, such as G2G (Shi *et al.* 2020), GraphRetro (Somnath *et al.* 2021), and RetroXpert (Yan *et al.* 2020) are also demonstrated in Table 1. Furthermore, the results of RetroCaptioner are also comparable to those motif-based and two-step retrosynthesis models while offering improved efficiency, such as MARS Liu *et al.* (2024) and Graph2Edits Zhong *et al.* (2023) as demonstrated in Supplementary Table S3.

**Table 1.** Top-k accuracy (%) for retrosynthesis prediction on USPTO-50k.^a^

Model	Top-k accuracy (%)							
	Reaction class known				Reaction class unknown			
	1	3	5	10	1	3	5	10
Transformer (Vaswani *et al.* 2017)	57.1	71.5	75.0	77.7	42.4	58.6	63.8	67.7
Tied Transformer (Kim *et al.* 2021)					47.1	67.1	73.1	76.3
Aug. Transformer (Tetko *et al.* 2020)					48.3	–	73.4	77.4
SCROP (Zheng *et al.* 2019)	59.0	74.8	78.1	81.1	43.7	58.6	63.8	68.7
GTA (Seo *et al.* 2021)					51.1	67.6	74.8	81.6
GET (Mao *et al.* 2021)	57.4	71.3	74.8	77.4	44.9	58.8	62.4	65.9
RetroExplainer* (Wang *et al.* 2023)	40.5	49.6	52.6	54.3				
MEGAN (Sacha *et al.* 2021)	60.7	82.0	87.5	91.6	48.1	70.7	78.4	86.1
G2G (Shi *et al.* 2020)	61.0	81.3	86.0	88.7	48.9	67.6	72.5	75.5
RetroXpert (Yan *et al.* 2020)	62.1	75.8	78.5	80.9	50.4	61.1	62.3	63.4
GraphRetro (Somnath *et al.* 2021)	63.9	81.5	85.2	88.1	53.7	68.3	72.2	75.5
RetroPrime (Wang *et al.* 2021)	64.8	81.6	85.0	86.9	51.4	70.8	74.0	76.1
Retroformer (Wan *et al.* 2022)	64.0	82.5	86.7	90.2	53.2	71.1	76.6	82.1
Graph2SMILES (Tu and Coley 2022)					52.9	66.5	70.0	72.9
RetroCaptioner (Ours)	**67.2**	**86.0**	**90.3**	**93.4**	**54.3**	**76.3**	**82.6**	**88.1**

a Result marked with an “*” in the table indicates the performance obtained from rerunning the model. The best performance is in bold.

As summarized in Table 1, with the exact matched accuracy (see Section 2 for detail), when the reaction class is known, RetroCaptioner achieves the best performance in all metrics, with a top-1 accuracy of 67.2%, top-3 accuracy of 86.0%, top-5 accuracy of 90.3%, and top-10 accuracy of 93.4%. RetroCaptioner significantly improves the prediction performance by over 10% compared to the vanilla Transformer model (Vaswani *et al.* 2017), which encodes molecular SMILES only. Specifically, RetroCaptioner enhanced the top-1, -3, -5, and -10 accuracy by 10.1%, 14.5%, 15.3%, and 15.7%, respectively. In addition, RetroCaptioner outperforms the two-step models RetroPrime (Wang *et al.* 2021), demonstrating that our model efficiently and accurately captures chemical information within one-step learning. Furthermore, our model surpasses all graph-enhanced Transformer models, indicating the superiority of our optimal graph representation and fusing strategy. For instance, compared to GET (Mao *et al.* 2021), RetroCaptioner increased top-1 accuracy by 9.8% and top-10 by 16%. In comparison to Retroformer (Wan *et al.* 2022), our model achieves improvements of 3.2% in top-1, 3.5% in top-3, 3.6% in top-5, and 3.2% in top-10 accuracy. With reaction class unknown, our model achieves the best performance among top-1 to 10 accuracy, a 54.3% top-1, 76.3% top-3, and 88.1% top-10 accuracy. Retroformer reaches state-of-the-art performance with top-1 accuracy. Moreover, compared with RetroExplainer (Wang *et al.* 2023), RetroCaptioner significantly boosts the average accuracy, achieving an improvement of around 30%.

### 3.4 Top-k SMILES validity

To validate that our model provides chemically plausible constraints could improve the SMILES validity. Experiment with Equation (8) to calculate the predicted SMILES validity. In our evaluation, we have excluded template-based methods. This decision is based on the fact that molecules constructed from templates, represented as SMILES, are inherently valid (Wan *et al.* 2022). Since SMILES generative and graph-based models are more likely to struggle with the validity issue (Chen *et al.* 2020, Wan *et al.* 2022, Wang *et al.* 2023).

As shown in Table 2, RetroCaptioner achieved the best performance and surpassed 90% in accuracy across all evaluation metrics. RetroCaptioner improved the top-10 validity by 24.7% over the vanilla Transformer. Sequence (SMILES)-based retrosynthesis models (Wang *et al.* 2021, Wan *et al.* 2022), typically offer higher SMILES validity than graph-based models like Graph2SMILES (Tu and Coley2022). RetroCaptioner significantly outperformed Graph2SMILES, with improvements of 22.9% in top-10, 13.8% in top-5, and 8.2% in top-3 SMILES validity. Since a reaction center represents the atoms and bonds involved in the chemical transformation (Shi *et al.* 2020, Wan *et al.* 2022), the results show that RCaptioner captions help the model avoid errors in non-reactive regions, generating highly valid SMILES sequences.

**Table 2.** Top-k SMILES validity (%) for retrosynthesis prediction on USPTO-50k with reaction class unknown.

Model	Top 1	Top 3	Top 5	Top 10
Transformer (Vaswani *et al.* 2017)	97.2	87.9	82.4	73.1
Graph2SMILES (Tu and Coley 2022)	99.4	90.9	84.9	74.9
RetroPrime (Wang *et al.* 2021)	98.9	98.2	97.1	92.5
Retroformer (Wan *et al.* 2022)	99.2	98.5	97.4	96.7
**RetroCaptioner (Ours)**	**99.4**	**99.1**	**98.7**	**97.8**

The best performance is in bold.

### 3.5 Assessment on natural products like reactions

In the field of drug discovery, a significant 64.9% of small-molecule drugs that have received approval from the Food and Drug Administration (FDA) since 1981 are either Natural Products (NPs) or derivatives of NPs (Zheng *et al.* 2022, Lee *et al.* 2024). However, fewer than 30 000 NPs function either as substrates or products. Thus, despite successes in chemistry, computer-aided synthesis of biocatalytics remains underdeveloped (Finnigan *et al.* 2021). Considering the complexity of biosynthesis and the fact that the limited number of verified enzymatic reactions. Zheng *et al.* (2022) proposed the NP-Like organic reactions (USPTO_NPL) dataset including reactions with components similar to NPs from the organic chemistry dataset [see Zheng *et al.* (2022) for details].

We validate the effectiveness and robustness of RetroCaptioner on the USPTO_NPL dataset with exactly matched accuracy (see Section 2) to assess the ability of retrosynthesis models to learn the reaction pattern for the NPL reactions (Finnigan *et al.* 2021, Levin *et al.* 2022). To facilitate a clear comparison, we benchmarked our model against the leading sequence-based (Wan *et al.* 2022) and graph-based (Somnath *et al.* 2021) models, which are the two top-performing methods (see Table 1). As shown in Table 3, RetroCaptioner achieved the best performance in all top-k accuracy.

**Table 3.** Top-k accuracy (%) for retrosynthesis prediction on USPTO-NPL.

Model	Top 1	Top 3	Top 5	Top 10
Graphretro (Sacha *et al.* 2021)	28.0	38.3	41.5	44.1
Retroformer (Wan *et al.* 2022)	30.0	41.9	46.5	52.0
**RetroCaptioner (Ours)**	**31.7**	**46.6**	**51.8**	**59.1**

The best performance is in bold.

Specifically, RetroCaptioner outperformed the state-of-the-art graph-based model Graphretro (Wang *et al.* 2023) by 3.73%, 8.3%, 10.3%, 15.04% in top-1, top-3, top-5, and top-10 exact matched accuracy. In addition, the results show that RetroCaptioner exhibits robust performance across different scenarios, demonstrating greater potential for drug discovery compared to previous state-of-the-art methods.

### 3.6 Ablation experiment

We evaluated the contribution of various RetroCaptioner components by comparing them with several variants to evaluate our proposed modules within the Transformer-based architecture. These variants include (i) SMILES sequence with the Transformer evaluating the performance when relying solely on SMILES. (ii) Simple concatenates molecular graph and SMILES (SMILES-Graph) without cross-attention, to show the effectiveness of adding graph feature. (iii) A variant with cross-attention, without the RCaptioner module, showing the benefits of our proposed dual-view fusion module, termed W/o RCaptioner. (iv) RetroCaptioner without the data augmentation for the training set, named RetroCaptioner_w/o aug_.

The results summarized in Table 4 present the performance of model variants. The Transformer model using a uni-view feature with SMILES sequences achieved top-1 accuracy of 57.1%. Adding molecular graph features to create a dual-view feature improved this accuracy to 60.2%, showing the advantage of integrating sequential and structural information. Further improvement was observed with the dual-view fusion mechanism in the W/o RCaptioner variant, which raised the accuracy to 64.8%.

**Table 4.** Ablation comparison on retrosynthesis with reaction class known.

Setting	Modules				Top-k accuracy (%)			
	SMILES	Graph	Cross-Att	RCaptioner	1	3	5	10
Transformer	✓				57.1	71.5	75.0	77.7
SMILES-Graph	✓	✓			60.2	80.2	86.1	90.8
W/o RCaptioner	✓	✓	✓		64.8	83.5	87.5	90.9
RetroCaptioner_w/o aug_	✓	✓	✓	✓	65.4	83.7	88.3	91.1
**RetroCaptioner**	✓	✓	✓	✓	**67.2**	**86.0**	**90.3**	**93.4**

The best performance is in bold.

Significantly, RetroCaptioner outperformed all variants, highlighting the superiority of the RCaptioner and RetroCaptioner_w/o aug_ module. For instance, comparing with W/o RCaptioner, RetroCaptioner achieved accuracy improvements of 2.4% for top-1, 2.5% for top-3, 2.8% for top-5, and 2% for top-10. When only encoding with the SMILES sequence, RetroCaptioner enhanced the top-1 accuracy by 10.1%. In addition, against SMILES-Graph, it showed a 7.0% improvement. These findings not only emphasize the critical role of the cross-attention-based fusion strategy but also underscore the superiority of the RCaptioner module for retrosynthesis with the Transformer-based architecture.

### 3.7 Case study: RetroCaptioner for drug retrosyntheic

To demonstrate the practical utility of our RetroCaptioner in drug pathway planning, we conducted a case study that underscores its efficacy in refining synthetic strategies for pharmaceutical compounds. We specifically used iterative top-1 predictions from RetroCaptioner to pinpoint commercially available molecules from *eMolecules* (Chen *et al.* 2020), thereby facilitating more accessible and cost-effective synthetic routes. A prime example of this is protokylol, as illustrated in Fig. 2. Using RetroCaptioner, we were able to simplify a previously complex four-step synthesis into a streamlined two-step route, as detailed in Wang *et al.* (2023). This not only demonstrates the efficiency of RetroCaotioner but also its potential to significantly reduce synthesis time and resource expenditure in pharmaceutical manufacturing.

We conducted a thorough literature review and used the SciFinder engine, grounding our prediction results in established chemical knowledge (Durandetti *et al.* 1996, Neudörffer *et al.* 2011, Liu 2017). This rigorous validation process led us to identify exact matches or high-yield analogs for the proposed reactions. Specifically, we pinpointed three chemical reactions (*R*1, *R*2, *R*3) that effectively produce protokylol, with detailed supporting evidence presented in Supplementary Fig. S1. Our findings not only validate the predictive power of RetroCaptioner but also open new avenues for its application in streamlining drug development processes, potentially leading to more efficient and sustainable pharmaceutical manufacturing practices.

In the initial reaction, RetroCaptioner identified two intermediates (I and II) when protokylol was used as an input. The related evidence 1 (Neudörffer *et al.* 2011) represented as *R*1, highlighted in light blue, suggests consistent transformations among similar functional groups which are highlighted in pink. For the transition from Intermediate I and II to buyable molecules, RetroCaptioner simplified the process into one step, and identical reactions were confirmed within the Google Patents library (Liu 2017) and Durandetti *et al.* (1996), respectively. The first step in the predicted pathway involves a reaction suggested by RetroCaptioner, which is confirmed by a SciFinder document showing a 100% yield, aligning with established synthetic methods. The second step achieves a remarkable 97% yield and illustrates the ability of RetroCaptioner to identify high-yield reactions from the literature. The final step from the prediction of RetroCaptioner matches a documented reaction with a 34% yield, also suggesting a practical and previously vetted synthetic route. This result indicates the reliability of our predicted reaction despite some specific reactions lacking direct precedents.

## 4 Conclusion

In our study, we introduced RetroCaptioner, a novel approach for end-to-end retrosynthesis prediction. The proposed RCaptioner captures reaction transition information and simplifies two-stage processes into one-step learning, emphasizing reactive centers through contrastive learning and enhancing traditional attention mechanisms. RetroCaptioner offers practical and precise reactant predictions, surpassing other methods on USPTO-50k dataset. In line with most data-driven retrosynthesis models, RetroCaptioner is currently unable to provide granular details of chemical reactions, such as temperature, and time duration for specific steps. This limitation is mainly due to gaps in deep learning capabilities and the lack of comprehensive datasets. Addressing these challenges is crucial for advancing automated synthesis systems and is a major focus of our future research. In summary, we believe our study provides new insights into Transformer models, and we plan to explore multi-step retrosynthesis with RetroCaptioner and direct graph-based reactant generation.

## Supplementary Material

btae561_Supplementary_Data
