# Supplementary material for: RetroCaptioner: beyond attention in end-to-end retrosynthesis transformer via contrastively captioned learnable graph representation
Source: Bioinformatics. 2024 Sep 28;40(9):btae561. doi: 10.1093/bioinformatics/btae561 (PMC11520410; doi:10.1093/bioinformatics/btae561)
Supplement: btae561_Supplementary_Data [file btae561_supplementary_data.pdf]

Supplementary Materials

1 Implementation details

Supplementary Table 1: Atom and bond features

Feature	Description
Atom Feature	
Atom symbol	C, N, O etc.
Degree of the atom	$\{d \in \mathscr{Z}; 0 \leq d \leq 9\}$
Formal charge of the atom	$\{d \in \mathscr{Z}; -2 \leq d \leq 2\}$
Valency of the atom	$\{d \in \mathscr{Z}; 0 \leq d \leq 6\}$
Hybridization of the atom	$sp, sp^2, sp^3, sp^3d, sp^3d^2$
Number of associated hydrogens	0.1.3.4.5
Chirality	R. S. unspecified
Part of an aromatic ring	True, false
Bond Feature	
Bond type	Single, double, triple, aromatic, other
Conjugated	True, false
Part of a ring	True, false

Supplementary Table 2: Model variable dimensions and descriptions

Variable	Dimension	Description	Dimension value
$\mathbf{H}_s$	$L \times d$	Sequence encoder output	$L \times 256$
$\mathbf{h}_i^{init}$	$d_v \times 1$	Initial atom features	128×1
$\mathbf{p}_i^{init}$	$k \times 1$	Positional encoding features	20×1
$\mathbf{e}_{ij}^{init}$	$d_e \times 1$	Initial bond features	128×1
$\mathbf{H}_g$	$m \times d$	Final node embeddings	$m \times 256$
$\mathbf{E}$	$n \times d$	Final edge embeddings	$n \times 256$
$\mathbf{w}$	$m \times 1$	Node weights	$m \times 1$
$\mathbf{H}_w$	$m \times d$	Weighted graph embedding	$m \times 256$
$\mathbf{H}$	$L \times d$	Final output of encoder	$L \times 256$

2 SMILES alignment

SMILES alignment constitutes an additional learning task for RetroCaptioner (Wan *et al.*, 2022). Analogous to machine translation, the SMILES sequences of source and target molecules often exhibit partial alignment, as a significant portion of the molecules remains unchanged during the reaction (Tetko *et al.*, 2020). The node alignment between graphs (i.e., atom mapping) can be straightforwardly converted into token alignment between SMILES (Zhong *et al.*, 2022).

3 Data augmentation

We adopt the data augmentation techniques described by Zhong et al., (Zhong *et al.*, 2022) for training SMILES generative models. Specifically, we apply SMILES permutation to the product and order permutation to the reactants during the training phase of USPT0-50k. By enumerating different atoms as the root of SMILES, we generate multiple input-output pairs to enrich the training dataset. During inference, we provide several distinct SMILES representations of the same input to produce multiple sets of outputs. The final prediction is obtained by uniformly scoring these outputs.

4 Reaction center predictor

Accurate prediction of the reaction center is meaningful for retrosynthesis. In our framework, we decompose the prediction of response centers into two sub-tasks of nodes and edges prediction. The node and edge features ($\mathbf{H}_g$ and $\mathbf{E}$), obtained from MPGNN-PE, are employed in two Feed-Forward Networks (FFN) identified as the Atom RC Identifier and Bond RC Identifier.

$$P_{rc}(h_i) = \sigma(\mathrm{FFN}_{\mathrm{atom}}(h_i)) \tag{1a}$$

$$P_{rc}(e_{ij}) = \sigma(\mathrm{FFN}_{\mathrm{bond}}(e_{ij})) \tag{1b}$$

where h_i and e_{ij} are the embedding for node i and edge between node i and j, the scores $P_{rc}(h_i)$ and $P_{rc}(e_{ij})$ represent the likelihood of the node and edge in the graph being predicted as a reaction center.

5 Top-k accuracy for retrosynthesis prediction

For the task of single-step retrosynthesis predictions, we adopt the top-k (k $= 1, 3, 5, 10$) exact match accuracy to evaluate the retrosynthesis performance. The exact match accuracy is computed by comparing predicted reactants SMILES to the dataset's ground truth on the USPTO-50k benchmark. The result of our model RetroCaptioner compared to Graph-based two-step models are shown in Supplementary Table 3

Supplementary Table 3: Top-k Accuracy for retrosynthesis prediction

Model	Top-k accuracy (%)							
	Reaction class known				Reaction class unknown			
	1	3	5	10	1	3	5	10
MARS (Liu *et al.*, 2024)	66.2	85.8	90.2	92.9	54.6	76.4	83.3	88.5
DualTF (Sun *et al.*, 2021)	65.7	81.9	84.7	85.9	53.6	70.7	74.6	77.0
Graph2Edits (Zhong *et al.*, 2023)	65.7	**87.3**	**92.0**	**95.3**	52.7	**77.2**	**85.3**	**91.0**
**RetroCaptioner(Ours)**	**67.2**	86.0	90.3	93.4	**54.3**	76.3	82.6	88.1

6 Case study

References

Liu, J. *et al.* (2024). Mars: a motif-based autoregressive model for retrosynthesis prediction. *Bioinformatics*, **40**(3), btae115.

Sun, R. *et al.* (2021). Towards understanding retrosynthesis by energy-based models. *Advances in Neural Information Processing Systems*, **34**, 10186–10194.

Tetko, I. V. *et al.* (2020). State-of-the-art augmented nlp transformer models for direct and single-step retrosynthesis. *Nature communications*, **11**(1), 5575.

Wan, Y. *et al.* (2022). Retroformer: Pushing the limits of end-to-end retrosynthesis transformer. In *International Conference on Machine Learning*, pages 22475–22490. PMLR.

Zhong, W. *et al.* (2023). Retrosynthesis prediction using an end-to-end graph generative architecture for molecular graph editing. *Nature Communications*, **14**(1), 3009.

Zhong, Z. *et al.* (2022). Root-aligned smiles: a tight representation for chemical reaction prediction. *Chemical Science*, **13**(31), 9023–9034.

<table>
<tr><th>RetroCaptioner Predicted Reactions</th><th>SCIFinder Document Reactions</th></tr>
<tr><td>Step1:

NCC(O)c1ccc(O)c(O)c1 +
CC(=O)Cc1ccc2c(c1)OCO2 ->
CC(Cc1ccc2c(c1)OCO2)NCC(O)c1ccc(O)c(O)c1</td><td>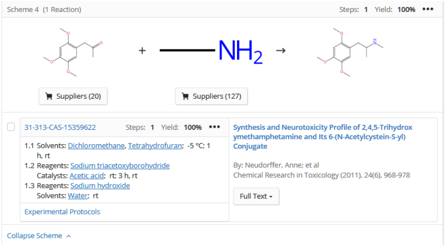
</td></tr>
<tr><td>Step2:

NCC(=O)c1ccc(O)c(O)c1 -> NCC(O)c1ccc(O)c(O)c1</td><td>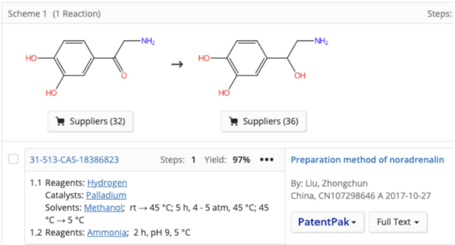
</td></tr>
<tr><td>Step3:

Brc1ccc2c(c1)OCO2 + CC(=O)CCl ->
CC(=O)Cc1ccc2c(c1)OCO2</td><td>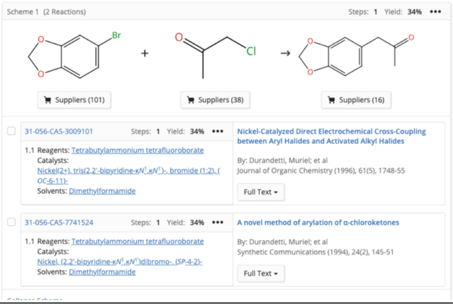
</td></tr>
</table>

Supplementary Figure 1: RetroCaptioner predicted reactions and documented reactions from SciFinder.
